# Single Nucleotide Variants in Transcription Factors Associate More Tightly with Phenotype than with Gene Expression

**DOI:** 10.1371/journal.pgen.1004325

**Published:** 2014-05-01

**Authors:** Priya Sudarsanam, Barak A. Cohen

**Affiliations:** Department of Genetics and Center for Genome Sciences and Systems Biology, Washington University School of Medicine, St. Louis, Missouri, United States of America; Georgia Institute of Technology, United States of America

## Abstract

Mapping the polymorphisms responsible for variation in gene expression, known as Expression Quantitative Trait Loci (eQTL), is a common strategy for investigating the molecular basis of disease. Despite numerous eQTL studies, the relationship between the explanatory power of variants on gene expression versus their power to explain ultimate phenotypes remains to be clarified. We addressed this question using four naturally occurring Quantitative Trait Nucleotides (QTN) in three transcription factors that affect sporulation efficiency in wild strains of the yeast, *Saccharomyces cerevisiae*. We compared the ability of these QTN to explain the variation in both gene expression and sporulation efficiency. We find that the amount of gene expression variation explained by the sporulation QTN is not predictive of the amount of phenotypic variation explained. The QTN are responsible for 98% of the phenotypic variation in our strains but the median gene expression variation explained is only 49%. The alleles that are responsible for most of the variation in sporulation efficiency do not explain most of the variation in gene expression. The balance between the main effects and gene-gene interactions on gene expression variation is not the same as on sporulation efficiency. Finally, we show that nucleotide variants in the same transcription factor explain the expression variation of different sets of target genes depending on whether the variant alters the level or activity of the transcription factor. Our results suggest that a subset of gene expression changes may be more predictive of ultimate phenotypes than the number of genes affected or the total fraction of variation in gene expression variation explained by causative variants, and that the downstream phenotype is buffered against variation in the gene expression network.

## Author Summary

There have been major efforts in the study of human disease to identify genetic polymorphisms that cause changes in gene expression. The assumption underlying these studies is that gene expression changes will be responsible for the disease. However, it is unclear if we can predict how a polymorphism affects the variation in disease based on the extent to which it explains variation in gene expression. We have taken advantage of four genetic polymorphisms that affect the ability of budding yeast cells to form spores. The variants were identified in naturally occurring strains, subject to natural selection pressures in the wild, and not from lab strains. These variants lie in factors that control gene expression, which gives us power to compare how the polymorphisms affect variation in both gene expression and the downstream phenotype. We find that the amount of variation in gene expression explained by the variants does not correlate with the amount of variation observed in spore formation, which has implications for studies that attempt to infer the effect of a polymorphism on phenotypic variation by studying its effect on gene expression variation.

## Introduction

Mapping the loci that control quantitative variation is a crucial step towards understanding complex disease [1]–[3]. Genome-wide association studies (GWAS) have shown that a large proportion of human disease-risk alleles consist of non-coding variants [4]. Since alterations in transcriptional regulation can drive disease states, there have been extensive studies to map eQTL, the genetic variants responsible for variation in gene expression [5]–[10] (for reviews, see [11]–[14]). Finding eQTL is now a widely accepted strategy for identifying new variants that potentially affect phenotype [15], for screening GWAS alleles to find those that affect disease risk by altering transcription [16], and for uncovering the molecular pathways underlying disease [17]. These studies make a distinction between *cis*-eQTL (genetic variants that affect the expression of physically linked genes) and *trans*-eQTL (variants that are physically unlinked from their target gene) [18]. *cis*-eQTLs also have effects in *trans* on unlinked genes that are downstream targets of the gene linked to the *cis*-eQTL. A large amount of effort is now directed towards the identification and analysis of eQTL. However, it remains extremely difficult to identify the precise nucleotide variant/s responsible for the changes in gene expression or phenotype, even in model organisms.

eQTL studies rely on an assumption that an unknown subset of the transcriptional changes in the target genes of the eQTL are responsible for the downstream disease phenotype. *cis*-eQTL that affect transcription factors are considered particularly interesting as they may identify the transcriptional program involved in the disease. However, despite numerous studies linking GWAS and eQTL results [16], [17], [19], fundamental questions remain about how a variant's effect on gene expression relates to its effect on phenotype. It is unclear if the amount of gene expression variation explained by an eQTL correlates with the amount of phenotypic variation it explains. In addition, it remains to be established if *cis*-eQTL play a more significant role in controlling gene expression variation compared to *trans*-eQTLs. The best way to address these questions would be to compare the effects of a set of variants that are responsible for changes in both gene expression and the ultimate phenotype.

Our lab has been studying the genetic variation responsible for the differences in sporulation efficiency in natural populations of *Saccharomyces cerevisiae* (*S. cerevisiae*) [20]. In the presence of nitrogen and non-fermentable carbon sources, diploid *S. cerevisiae* cells face a cell fate decision that involves a switch from fermentation to aerobic respiration and the cessation of mitosis followed by the initiation of meiosis [21]–[23]. Sporulation efficiency is defined as the percentage of cells in a culture that form meiotic spores, and is a highly heritable, complex trait [20], [24]–[26]. We have identified the exact nucleotide variants responsible for most of the variation in sporulation efficiency between a natural oak tree isolate (YPS606) and a vineyard strain (BC187) [27]. The oak tree isolate sporulates at 100% efficiency while the vineyard strain sporulates at 3.5% under sporulating conditions [27], [28]. By swapping the causative nucleotides in the vineyard background for the oak nucleotide variants, we generated an isogenic panel of vineyard strains that have completely identical genomes except at the causative variants [27]. Here, we describe the use of this allele replacement strain panel to study the primary question posed above: What is the relationship between the effect of causative nucleotides on the variation in gene expression and in phenotype?

There are four quantitative trait nucleotides (QTNs) in three genes (*IME1*, *RME1* and *RSF1*) that are responsible for most of the differences in sporulation efficiency between the oak and vineyard strains [27]. The four QTN consist of two non-coding and two coding variants. The non-coding regions of *RME1* (*RME1nc* - *RME1(indel-308A)*) and *IME1* (*IME1nc* - *IME1(A-548G)*) contain one causative variant each, implying that changes in *RME1* and *IME1* expression may be responsible for the differences in sporulation efficiencies between the parent strains. The remaining two QTN are coding variants in *IME1* (*IME1c* - *IME1(L325M)*) and *RSF1* (*RSF1c* - *RSF1(D181G)*). Strikingly, the three QTN-containing genes are either known (*IME1* [29], [30] and *RME1* [31]) or putative (*RSF1* [32], [33]) transcription factors. Given their role in transcriptional regulation, it is reasonable to assume that the four sporulation QTN affect phenotype through changes in gene expression. The allele replacement panel is isogenic at all loci, except for the causative variants. Since the sporulation QTN are the only genetic variants in the panel, they must be responsible for all reproducibly observed gene expression variation among the panel strains. Consequently, the sporulation alleles are nucleotide variants responsible for the variation in phenotype (QTN) as well as variation in gene expression (eQTN).

We present the results of a study in which we measured the effects of individual single-nucleotide variants on both gene expression and sporulation efficiency in a controlled setting. Since the QTN underlying variation in sporulation efficiency reside in transcription factors, and have been swapped individually and in all combinations into a clean background, our experiment represents a rigorous test of the relationship between the effect of a variant on gene expression and on the ultimate phenotype. Our analysis reveals that 1) the amount of variation in gene expression explained by a polymorphism is not always correlated with the amount of phenotypic variation explained by that same polymorphism, 2) genetic interactions between variants are responsible for a larger proportion of gene expression variability than phenotypic variability, and 3) that alleles that change either the level or activity of a transcription factor affect expression variation of the same genes to different extents. We also find that while the allele replacement panel displays extensive variation in gene expression, the downstream phenotype is largely buffered from the variation in the upstream transcriptional network.

## Results

### Single QTN are responsible for variation in both gene expression and sporulation efficiency

To explore the relationship between genetic variation, gene expression and phenotype, we utilized a panel of sixteen isogenic strains in the vineyard background. The panel was generated by swapping causative vineyard nucleotides with their oak allele counterparts [27]. This panel includes the vineyard parent, the “vineyard converted” strain that has all four oak QTN in place of the vineyard alleles, as well as strains with all possible combinations of oak and vineyard alleles at the four QTN. Using conditions which differed slightly from those in *Gerke et al* [27] (see Materials and Methods), we first measured the sporulation efficiencies of the allele replacement strains to quantify the effects of the QTN on sporulation efficiency under these conditions (Table S1). We assessed the effect of genotype on sporulation efficiency by building a linear model of the effects of the four QTN on sporulation efficiency (Table S2). The analysis of variance shows that the allelic status of the QTN explains 98% of the differences in sporulation efficiencies between the strains in the panel (Table 1). 93% of the variance in sporulation efficiency is due to a simple linear combination of the individual (main or additive) effects of the four vineyard QTN alleles (Table 1). The variation in sporulation efficiency explained by the main effects of the vineyard alleles of *RME1nc*, *RSF1c* and *IME1c* is almost equal while the vineyard allele of *IME1nc* explains a smaller but significant amount. An additional small but significant amount of variance (5%) can be explained by the genetic interactions between the vineyard alleles. The small number of significant interaction parameters indicates that a simple additive model of the main effects between the four QTN explains almost all the variation in the phenotype under these conditions.

**Table 1:** Analysis of variance (ANOVA) table of sporulation efficiencies in allele replacement strains.

Source of Variation	Df	Sum of Squares	Mean Square Error	F value	P value	Fraction of variance explained (%)
*RME1nc*	1	7702.0	7702.0	864.2	**<2e-16**	34.32
*RSF1c*	1	4517.0	4517.0	506.8	**<2e-16**	20.13
*IME1c*	1	7212.0	7212.0	809.3	**<2e-16**	32.13
*IME1nc*	1	1459.1	1459.1	163.7	**<2e-16**	6.50
RME1nc*RSF1c	1	293.7	293.7	33.0	**6.22e-07**	1.31
RME1nc*IME1c	1	134.3	134.3	15.1	**0.0003**	0.60
RME1nc*IME1nc	1	84.6	84.6	9.5	**0.0034**	0.53
RSF1c*IME1c	1	118.6	118.6	13.3	**0.0007**	0.38
RSF1c*IME1nc	1	32.9	32.9	3.7	0.6062	0.15
IME1c*IME1nc	1	124.6	124.6	14.0	**0.0005**	0.56
RME1nc*RSF1c*IME1c	1	161.4	161.4	18.1	**9.59e-05**	0.72
RME1nc*RSF1c*IME1nc	1	4.4	4.4	0.5	0.4865	0.02
RME1nc*IME1c*IME1nc	1	136.3	136.3	15.3	**0.0003**	0.61
RSF1c*IME1c*IME1nc	1	34.6	34.6	3.9	0.0546	0.15
RME1nc*RSF1c*IME1c* IME1nc	1	0.1	0.1	0.01	0.9230	0.00031
*Residuals*	48	427.8	8.9			1.91

All experiments were performed in the vineyard strain background. The four sporulation QTN are: *RME1nc*: *RME1(indel-308A)*, *RSF1c*: *RSF1(D181G)*, *IME1c*: *IME1(L325M)*, *IME1nc*: *IME1(A-548G)*. The source of variation in sporulation efficiency is due to the effect of changing the genotype from the oak to the indicated vineyard allele in the vineyard converted strain (all four oak alleles in the vineyard background). P-values≤0.05 are in bold. ***Fraction of variance explained: Additive Factors = 93.08%; Interaction Factors = 5.02%***.

We next measured the effect of each QTN on global-expression profiles during the cell fate decision phase when all three genes are active. *RSF1* is required for transcription of mitochondrial genes [32] and respiration is known to be required for Ime1 expression and meiosis [34]. In addition, *RME1* [31] and *IME1* [30], [35] control some of the critical transcriptional changes during this phase. *IME1* expression is induced rapidly after the switch to sporulation medium [35]. We showed previously that differences between the oak and vineyard strains in making the decision to sporulate occur very early after the switch to non-fermentable carbon, before meiotic DNA synthesis [20]. We, therefore, used RNA-Seq [36] to measure global mRNA expression-profiles in all sixteen strains in the panel after two hours in sporulation medium, before meiotic DNA replication begins. We surmised that the causative QTN would be active during this period and that the differences in gene expression between the strains at this time point would be linked to the differences in sporulation efficiencies. We obtained good reproducibility between the biological replicates (the range of mean Pearson's correlation coefficients for pair-wise comparisons between replicates of each strain was 0.86–0.93). The coefficient of variance, CV, (standard deviation/mean), for the biological replicates is a measure of the variance in our measurements. The CV for gene expression (median CV = 0.15) is slightly greater than the CV for sporulation efficiency (median CV = 0.076) but is consistent with reports from previous RNA-Seq experiments [37], [38].

**Figure 1:**
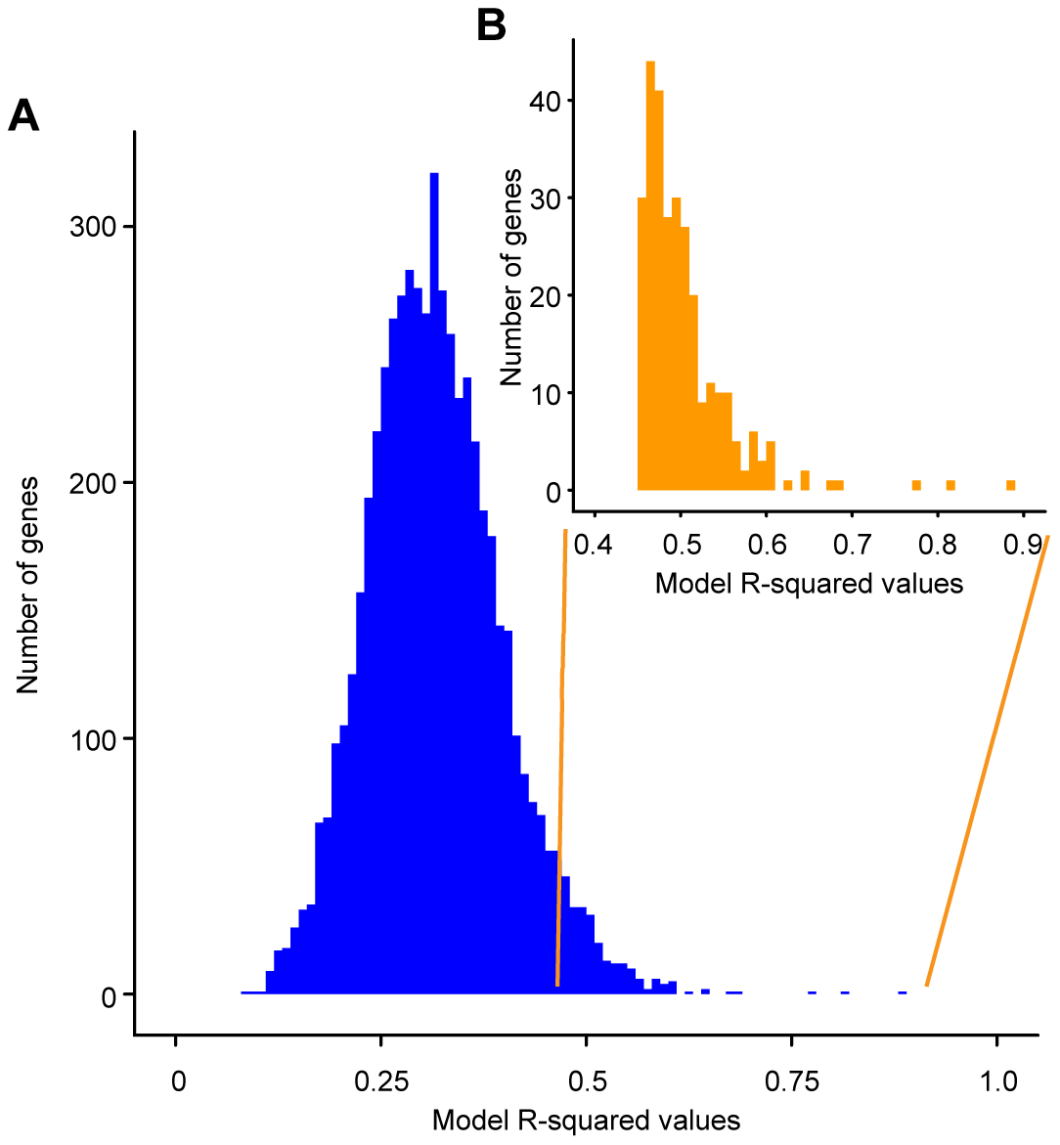
Histogram of R-squared values obtained for the linear models describing the effect of genotype on the expression of individual genes. The R-squared values obtained are on the x-axis and the numbers of gene expression models with the particular R-squared values are on the y-axis. A) Histogram of the R-squared values for all 5792 genes in the *S. cerevisiae* genome. B) Histogram of the R-squared values for the 289 significant gene expression models *(inset)*. Significant models have an unadjusted model p-value≤0.006.

We assessed the effects of the QTN on the expression of each gene in the genome by regressing genotype on gene expression patterns across the sixteen strains in the panel. After removing the effect of day-to-day experimental variation (see Materials and Methods), we applied a linear model framework to assess how much of the variation in the expression of each gene could be explained by the allelic status of the sporulation QTN (Table S3). After correcting for multiple hypothesis testing, we obtained 289 significant gene-specific models (∼5% of the genome) in which gene expression was significantly affected by the allele status of the QTN (Figures 1 & S1).

Within these 289 genes, the genetic status of the QTNs explains 45–88% of the observed variation in expression (median 49%) (Figure 1 (inset), Table S4). The best model of gene expression (for *URC2*, a putative Zn(II)Cys6-containing transcription factor [39]) explains 88% of the variance in this gene's expression. These results stand in stark contrast to the model of sporulation efficiency, which explains 98% of the variation in this phenotype. The median variance explained by the polymorphisms depends on the exact FDR we chose in our analysis (a lower FDR would yield a higher median variance explained). However, for any FDR threshold, the gene expression models are always less predictive than the sporulation model. Applying a similar linear model framework to log-transformed expression counts did not increase the gene expression variance explained by the QTN (Figure S2) and, therefore, we analyzed the models using untransformed gene expression counts. These results suggest that the statistical relationship between QTN and phenotype is simpler than the link between eQTN and gene expression.

### Balance between main and interaction effects on the variation in gene expression versus sporulation efficiency

**Figure 2:**
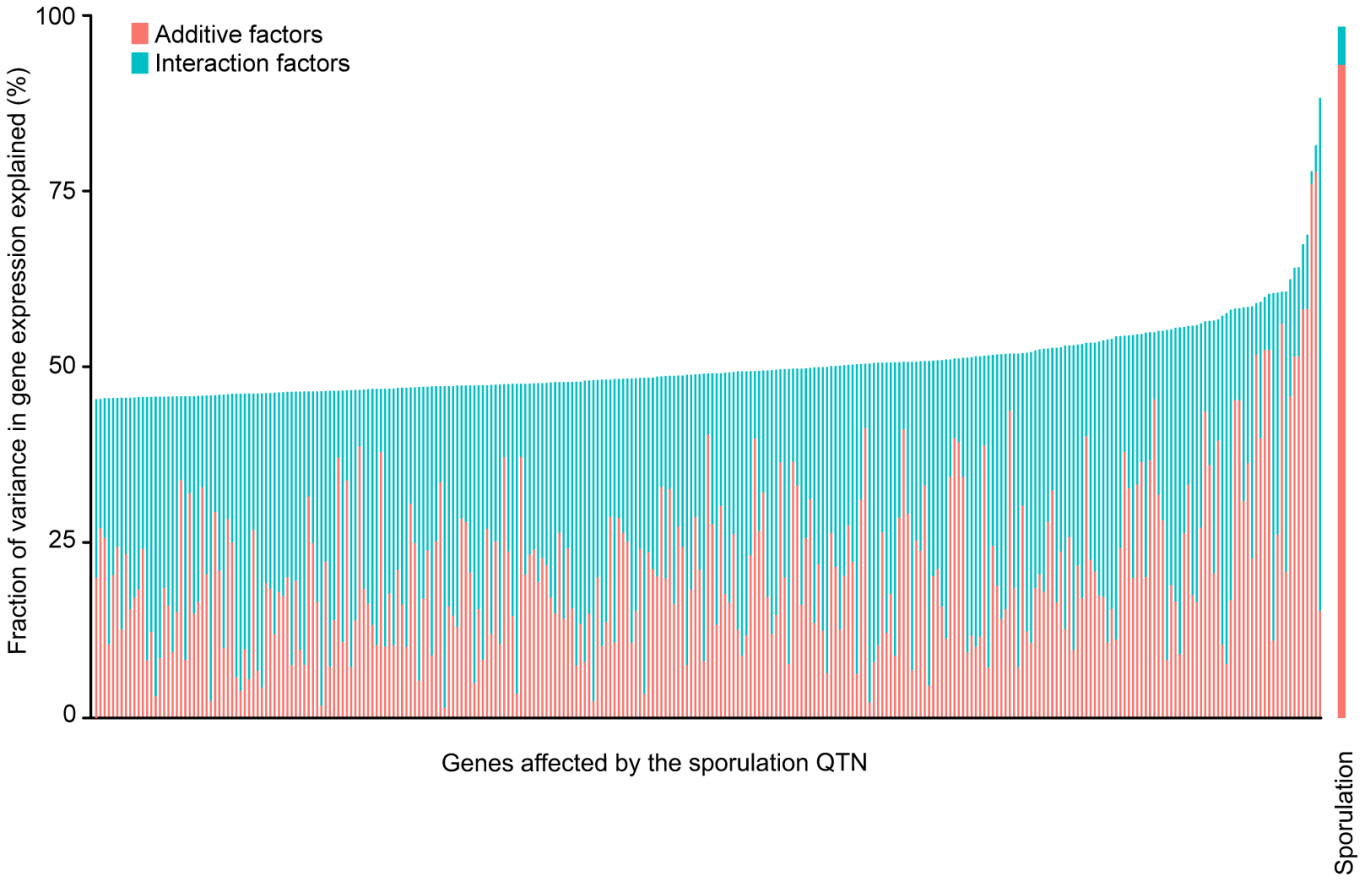
Fraction (%) of sporulation and gene expression variance explained by main *(pink)* and interaction effects *(cyan)* of all four sporulation QTN together. Only the 289 ORFs with significant gene expression models are shown. The ORFs are ordered by fraction of total variance explained in the full model. Each column represents the amount of variation in gene expression explained for a given ORF. The last column represents the fraction of sporulation efficiency variance explained by the QTN. Only the significant ANOVA factors in both the sporulation efficiency and gene expression models were considered to calculate the fraction of variance explained by main and interaction effects (f-statistic p-value<0.1).

Genetic interactions between the QTN account for a large fraction of the variation in gene expression. We found that all four QTN play a role in the expression of most of the 289 significantly affected genes, either through main or interaction effects (Tables 2 & S3). As *RME1*, *IME1* and *RSF1* act at similar points in the sporulation network [23], [33], [34], it is not surprising that interactions between the alleles explain a major portion of the variation in gene expression (Figure 2). Main and interaction effects explain almost equal amounts of the variation in gene expression, which stands in contrast to the model for sporulation efficiency, in which main effects explain the vast majority of the variation in phenotype. The median variance in gene expression explained by main effects of the QTN across all 289 genes is 20% and by the interaction effects is 29.7%. Only a small fraction of the genes (26/289) show the additive-interaction balance observed in the sporulation model where main effects account for over 90% of the explained expression variance. These genes include *RIM4* (a known target of Ime1 [40]), *RME1* itself, and *PRD1* (a zinc metalloendopeptidase that is involved in the degradation of mitochondrial proteins [41]). Our results show that, while complex interactions between the QTN drive most of the variation in gene expression patterns, additive effects of the QTN account for most of the variation in sporulation efficiency under the conditions tested here. Given the significant differences between the explanatory power of the gene expression models and the sporulation efficiency model, our results suggest that the downstream phenotype is robust to expression variation in the network.

**Table 2:** Summary of sporulation QTN effects on gene expression.*

Sporulation QTN	Number of ORFs with significant main &/or interaction effects	Number of ORFs with significant main effect	Fraction of ORFs where allele has significant main effect (%)	Median total variance explained (%)	Median additive effect (%)	Median interaction effect (%)
*RME1nc*	264	92	35	15	0	12
*RSF1c*	287	205	71	29	8.5	16
*IME1c*	275	97	35	17	0	14
*IME1nc*	273	134	49	19	0	16

* *Results shown are for the 289 genes with significant gene expression models.*

**Total Variance Explained:** For each gene with a significant model (model p-value≤0.006), fraction of total variance explained by all significant effects of the allele in the ANOVA table (F-test p-value of effect <0.1).

**Main (Additive) Effect:** For each gene with a significant model (model p-value≤0.006), fraction of total variance explained by significant main effect of the allele in the ANOVA table (F-test p-value of effect <0.1).

**Interaction Effect:** For each gene with a significant model (model p-value≤0.006), fraction of total variance explained by all significant interaction effects of the allele in the ANOVA table (F-test p-value of effect <0.1).

**Figure 3:**
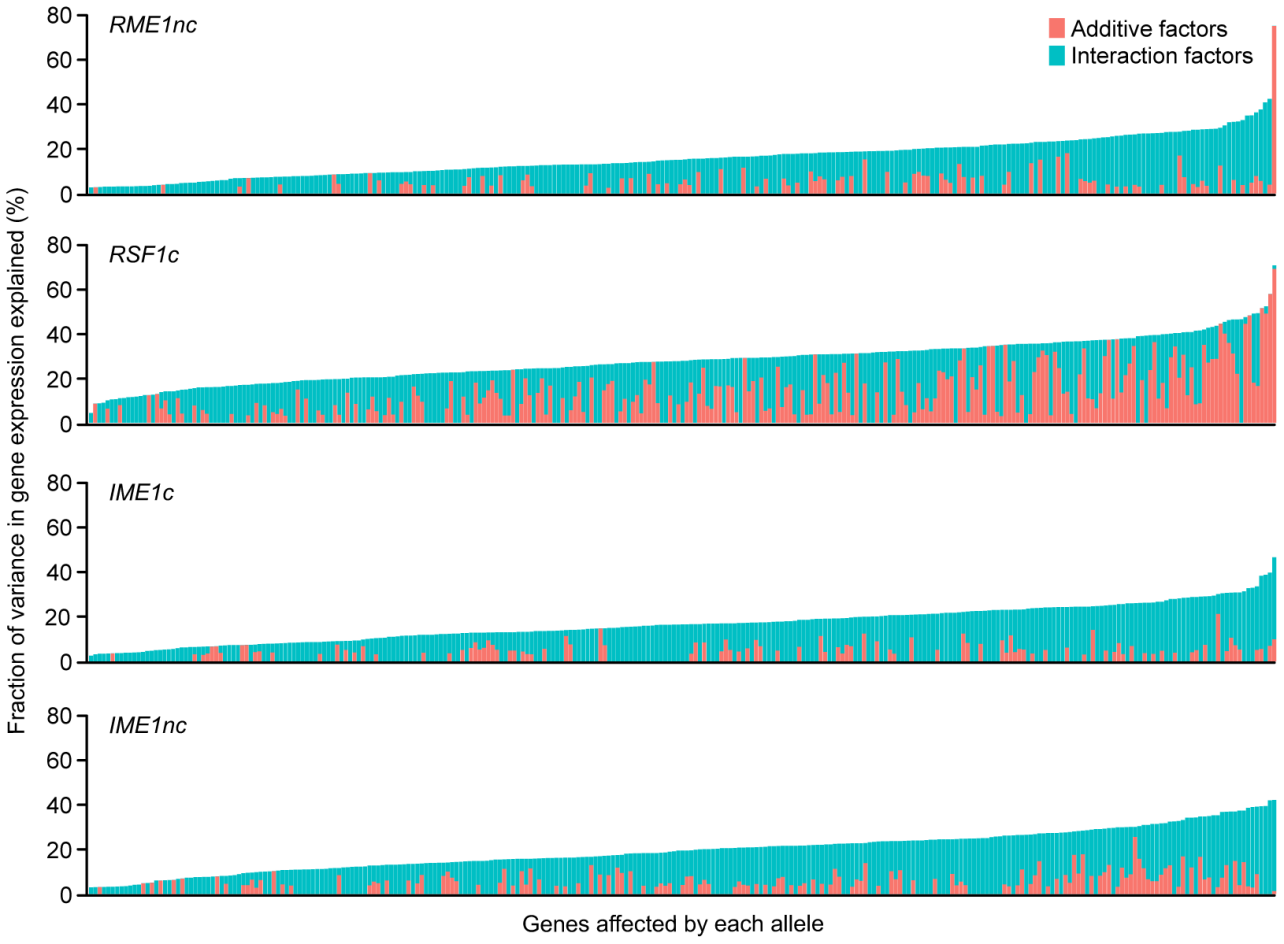
Fraction (%) of gene expression variance explained by main *(pink)* and interaction effects *(cyan)* of each of the four sporulation QTN. The QTN effect on the 289 ORFs with significant gene expression models is shown. The ORFs are ordered by fraction of total variance explained in the full model. Plot includes only those models in which the fraction of gene expression variance explained by the particular QTN is greater than zero. Each column represents the amount of variation in gene expression explained for a given ORF. Only the significant ANOVA factors (f-statistic p-value<0.1) for each QTN were considered.

We also found that the balance between main and interaction effects on the variation in gene expression was different for different QTN (Figure 3). *RSF1c*'s role in controlling expression variation was primarily through its main effects while *RME1nc* and both *IME1* alleles exerted their influence on expression variation primarily through interactions with the other alleles. These results are not surprising as *RME1* and *IME1* act at the same point in the sporulation transcriptional network [23] with Rme1 binding directly to the promoter of *IME1* [31].

### Comparison of the effect of QTN on variation in gene expression and sporulation

**Figure 4:**
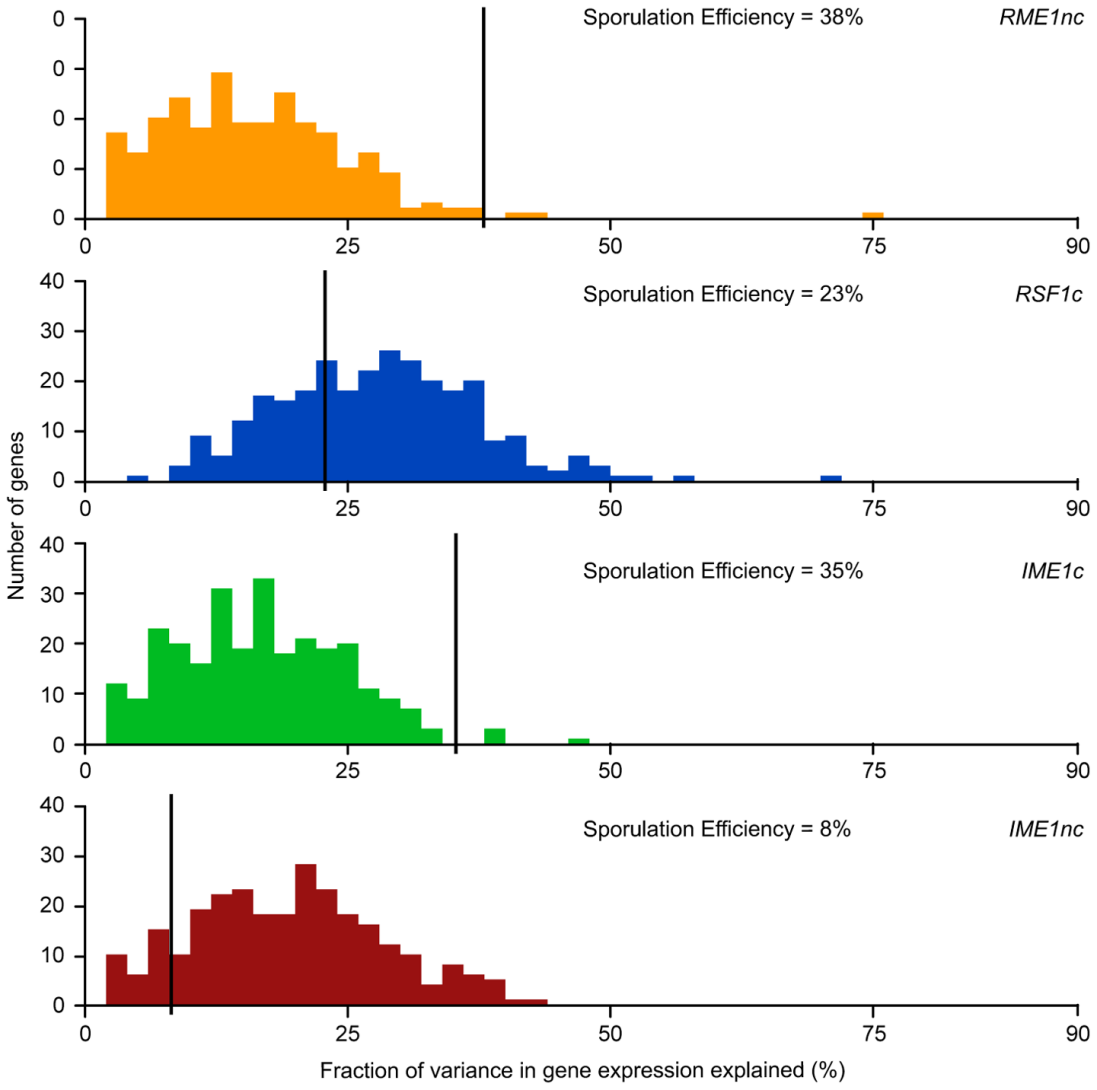
Histogram of total fraction (%) of gene expression variance explained by each QTN. For each QTN, total fraction of gene expression variance explained *(x-axis)* is calculated by the sum of the significant main and interaction terms. The number of significant gene expression models with the given fraction is plotted on the y-axis. Only the significant ANOVA factors (f-statistic p-value<0.1) for each QTN were considered. The black line represents the fraction of the variation in sporulation efficiency that is explained by the given QTN (also listed in each figure).

We next asked whether the fraction of variation in gene expression explained by sporulation QTN was similar to that explained for sporulation efficiency. We found that the proportion of gene expression variation explained by the QTN was not predictive of the explanatory power in the sporulation efficiency model. *RSF1c* controls the variation in expression of a large number of genes. It affects the expression of almost all of the 289 genes with significant expression models and explains a significant proportion of the variation of 71% of the target genes (205/287 genes) (Table 2). The main effect of *RSF1c* also explains the largest proportion of the variation in gene expression compared to the other three QTN (median variance explained by *RSF1c* main effect = 8.5%, Figure 3). However, it is surprising that, despite its significant role in gene expression, *RSF1c* does not have the largest role in explaining the variation in sporulation efficiency. The *RSF1c* allele explains 23% of the variation in sporulation efficiency as compared to *RME1nc* (38%) and *IME1c* (35%) (Table 1B, Figure 4). Little is known about *RSF1* except that it may be a transcriptional modulator of respiration [32] which is known to be required for sporulation in *S. cerevisiae* [34]. These results suggest that *RSF1* plays a significant role in the transcriptional cascade that initiates sporulation along with the known sporulation transcriptional regulators, *RME1* and *IME1*. However, it is also possible that, despite being responsible for a large fraction of the variation in gene expression, only a subset of *RSF1c*'s target genes affect sporulation efficiency. In contrast, *RME1nc* or *IME1c* may account for a greater proportion of the variation in the phenotype as more of their target genes may be directly involved in sporulation.

*RME1nc* and *IME1c* both explain a comparatively modest fraction of the variation in gene expression (Figure 4). The main effects of both alleles account for the expression variation of 35% of their targets (Table 2) but exert their influence primarily through interactions with the other QTN (Figure 3). As stated before, this is not surprising as Rme1 and Ime1 act at the same point of the transcriptional cascade [23] and *RME1* is a known repressor of *IME1* expression [31]. The expression of *RME1* itself is a notable exception. The main effect of *RME1nc* explains 75% of the variation in *RME1* expression (Table 3). The expression of *RME1* is almost bimodal with increased expression in strains containing the *RME1nc* oak allele and reduced expression in the presence of the vineyard allele. These results are striking given the role of the two QTN on the variation in sporulation efficiency. The main effects of *RME1nc* and *IME1c* explain a large proportion of the variation in sporulation efficiency (Table 1, Figure 4). However, their role in controlling gene expression variation is not as significant as *RSF1c* and occurs primarily through interactions with the other alleles (Figure 3). These results, again, highlight the differences between the QTN in their control of gene expression and sporulation efficiency variation.

**Table 3:** Gene expression models for the genes containing the sporulation QTN.

Gene	Gene Expression Model*	Multiple R-squared#	F-test p-value
*RME1*	*E_RME1_* = −116.1+256 *RME1nc_V_*	0.8	1.9e-12
*IME1*	*E_IME1_* = 394.6−506 *RSF1c_V_*−223 *IME1nc_V_*	0.6	1.8e-05
*RSF1*	*E_RSF1_* = −4	0.3	0.2

*E_<gene>_ represents the residual expression of the particular gene after the effect of experimental variation is removed. The first term in the model (intercept) is the mean residual expression of the gene in the vineyard strain with all four oak QTN (Vineyard OOOO). Each subsequent term in the model represents the gene expression effect of replacing the oak allele of the particular QTN with the vineyard allele in the Vineyard OOOO strain (−/+ indicates direction of effect). Only significant terms in the model are shown Pr(>|t|)<0.1.

# R-squared value obtained from applying the full model containing all possible main and interaction effects between the four sporulation QTN.

*IME1* is considered the primary regulator of the sporulation transcriptional cascade [30], [42]. However, the *IME1nc* allele does not explain as much of the variation in gene expression as *RSF1* (Table 2) possibly because *RSF1* acts earlier than *IME1* and affects both respiration and sporulation genes. Accordingly, *RSF1* is responsible for a significant proportion of the variation in *IME1* gene expression (Table 3) though it is unclear if it directly affects the transcription of *IME1*. Similar to *RME1* and the coding allele of *IME1*, *IME1nc* affects gene expression through genetic interactions with the other three alleles (Figure 3). The main effects of *IME1nc* explain the variation of a slightly larger number of genes than *IME1c* (134/273 genes) (Table 2). It is striking, therefore, that *IME1nc*, is responsible for the smallest proportion of the variation in sporulation efficiency (Figure 4) showing that a genetic variant such as *IME1nc* can cause significant changes in the variability of gene expression upstream in the network but play a modest role in the variation of the ultimate phenotype. Our results thus indicate that the proportion of variation in gene expression explained by a QTN is not predictive of the amount of phenotypic variation that it explains.

The number of eQTL targets has also been used to identify “hot spots” of regulatory activity that may be important for the disease phenotype [5], [10], [43], [44]. In addition, there has been some discussion that *trans*-eQTL are more likely to be eQTL “hot spots” than *cis*-eQTL as their effects may be more pleiotropic [45]. The oak and vineyard parental strains used in these studies also exhibit some pleiotropy as they differ in the size of the cells entering meiosis, the relative numbers of dyads, triads, and tetrads in fully sporulated cultures, and growth on non-fermentable carbon sources [20]. While we know that the *RSF1c* is not responsible for the growth differences of the parental strains on glycerol [27], it is possible that some of the sporulation QTN-dependent genes may influence these other phenotypes. All four of the eQTN studied here affect the expression variation of a large number of overlapping genes (Table 2), thereby, behaving as expression “hot spots”. As expected, the *cis*-eQTL affect the variation in gene expression of the linked genes (*RME1* and *IME1*) but also affect the variability of many genes in *trans*. We do not observe any consistent differences in the number of genes whose expression variation is affected by either the *cis*-eQTL (*RME1nc* and *IME1nc*) or the *trans*-eQTL (*RSF1c* and *IME1c*). We also do not find significant enrichment for any particular gene ontology (GO) category (*P.S & B.A.C, unpublished data*). More importantly, as described above, even though all four eQTN behave as “hot spots” for transcriptional changes, there are significant differences in the amount of downstream phenotypic variation that they control. The comparisons indicate that the number of genes affected, the balance between the additive-interaction effects in their control of expression variation and the fraction of gene expression variance explained are not predictive of the effect of the QTN on sporulation efficiency.

### Comparison of the *IME1* coding and non-coding QTN

**Figure 5:**
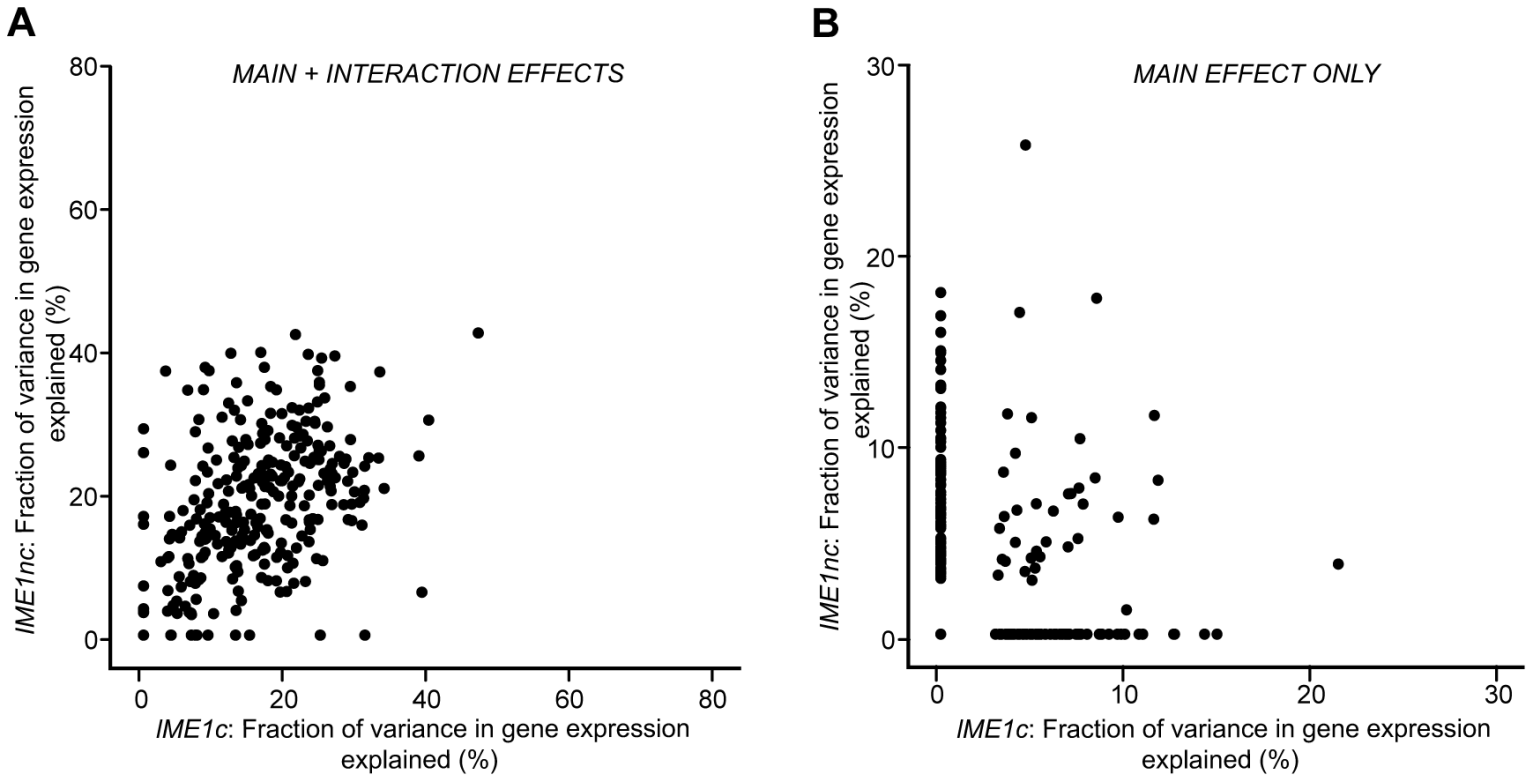
Scatter plot comparing the total fraction of variance explained by the *Ime1nc* and *Ime1c* alleles. a) Fraction of variance explained by main and interaction effects. b) Fraction of variance explained by main effects alone. For each QTN, fraction of expression variance explained is calculated by using the significant main and interaction terms of the QTN (f-statistic p-value<0.1). Results are shown for the 289 ORFs with significant gene expression models.

One striking result is the difference between the effects of the two *IME1* QTN on the variation in sporulation efficiency. The non-coding allele of *IME1*, *IME1nc*, affects the expression level of *IME1* and consequently, the amount of Ime1 protein. The coding allele of *IME1*, *IME1c*, probably affects the activity of Ime1 protein as it lies in a domain of Ime1 that is responsible for protein-protein interactions with Rim11 and Ume6 [30], two factors that are required for the initiation of sporulation. Given that both alleles occur in the same transcription factor, we investigated if their effects on the variation in gene expression matched their roles in controlling variation in sporulation efficiency. While the distributions of the effects on the variation in gene expression for the two alleles look very similar and they affect similar sets of genes (Figure 4), the *IME1c* allele explains a larger proportion of the variation in sporulation efficiency than *IME1nc* (Table 1). Closer inspection of the expression data revealed that while both alleles explained the expression variation of the same set of genes, the rank order of the amount of variance explained by each of the alleles is quite different (p-value<0.005, Wilcoxon rank sum test). In other words, the two *IME1* alleles both affect the same set of genes, but expression variation of specific genes is more or less sensitive to either the coding or non-coding allele. These differences can be seen by comparing the fraction of variance explained by the two *IME1* alleles in individual gene expression models. While the expression variation of most *IME1*-dependent genes is affected by both alleles when the full model is applied, the proportion of variance explained varies between the alleles (Figure 5a, correlation coefficient, r = 0.43). This difference between the alleles is magnified when only the variance explained by main effects is considered (Figure 5b). While there are a few genes where the main effects from both alleles affect a significant proportion of the variation, the expression variation of most of the dependent genes is affected primarily by only one or the other allele. The difference between subsets of genes in their sensitivity to either the level (*IME1nc*) or the activity (*IME1c*) of Ime1 manifests itself as a dramatic difference in the effects of the two *IME1* alleles on sporulation efficiency.

## Discussion

We have used a set of individual single nucleotide variants in known or putative transcriptional regulators that are causative for variation in sporulation efficiency to explore the relationship between genetic variants and their effects on gene expression and phenotype. The allele status of the QTNs explains almost all of the variation in sporulation efficiency but the median variation in gene expression explained is only 49%. In addition, variation in gene expression results from many interactions between the alleles while simple additive effects of the QTN explain most of the variation in sporulation efficiency. It is intriguing that gene expression varies more than the phenotype as the four QTN represent the sole genetic changes in the panel. Why might the QTN show a stronger correlation with sporulation efficiency than with expression variation, even though the QTN reside in transcriptional regulators? It is possible that our gene expression measurements are “noisier” than those of sporulation efficiency as RNA-Seq may be more sensitive in measuring variation in gene expression than the fluorescence measurements used to assess sporulation efficiency. It is also possible that experimental variation was introduced during sample preparation. We know that day-to-day variation in media conditions, oxygen levels, etc. can affect sporulation efficiency and expect that they would affect gene expression as well. We accounted for this variation by including the day of growth as a covariate in our gene expression models. However, it is possible that there is some additional unexplained gene expression variation even among strains grown on the same day.

The fact that genotype better explains sporulation efficiency than the “endo-phenotypes” of gene expression suggest that sporulation efficiency is buffered from changes in the transcriptional network. Developmental biologists have invoked the concept of “phenotypic robustness” to explain how body patterns remain invariant despite perturbations in the upstream gene regulatory network [46], . QTL mapping studies in *Arabadopsis* lines have also suggested that genetic variation in gene expression does not always manifest itself as phenotypic variation [48]. Phenotypic changes often require gene expression changes beyond certain thresholds. As long as transcriptional fluctuations do not cross the threshold, the phenotype does not vary. When transcription is tuned to be close to the threshold, variability in gene expression has been shown to be responsible for incomplete penetrance [49]. Conversely, surplus gene expression i.e. gene expression levels that are considerably higher than the threshold needed to cause phenotypic change, can result in “wild-type” phenotypes [50]. The fact that, in our conditions, main effects account for most of the variation in sporulation efficiency whereas allele interactions account for a significant, but much smaller amount of the phenotypic variation, suggests that the sporulation efficiency phenotype is buffered from the variation in the transcriptional network. The sporulation transcriptional cascade contains multiple points for feedback control [51] which probably impose several thresholds on gene expression levels. One obvious possibility is that cells only sporulate when the levels of the sporulation transcriptional activators are above a certain level. This also implies that, in properly powered studies, genotype will be more strongly associated with phenotype than with gene expression.

Our analyses of the relationship between gene expression variation and sporulation efficiency variation are based on expression measurements taken at a single time point. We chose to analyze the gene expression changes at this early stage of sporulation as the transcription factors containing the sporulation QTN exert their effects soon after the switch into sporulation medium. In addition, *Gerke et al.* [20] showed that the critical differences between the oak and vineyard parental strains also occur early in sporulation. Gene expression changes at later time points are likely to correlate better with sporulation efficiency, but this correlation will be driven by gene expression changes due to differences in the numbers of actively sporulating cells. Our expression measurements reflect the early gene expression changes in the decision to sporulate during the period when the QTN are active, not the downstream effectors of sporulation.

The main effects of the two *IME1* alleles, *IME1nc* and *IME1c*, play distinct roles in controlling the variation in gene expression, despite residing in the same transcription factor. Our results suggest that individual target genes are more dependent on either the level (*IME1nc*) or activity (*IME1c*) of Ime1. Ime1 binds its target promoters through Ume6, which encodes a DNA-binding protein [52]. Binding of Ime1 for Ume6 activates transcription of early-meiosis genes by displacing the repressive activities associated with Ume6 [30]. The *IME1c* allele probably affects the affinity of Ime1 for Ume6 or other co-factors as it lies in a domain of Ime1 that is responsible for protein-protein interactions with Rim11 and Ume6 [30]. Given this mode of action, the differences between the two *IME1* alleles suggest that changing the affinity of Ime1 to Ume6 or other co-factors has a different effect on *IME1*-dependent promoters compared to changing the concentration of Ime1. It is possible that Ime1 exhibits cooperativity at *IME1nc*-dependent genes but not at *IME1c*-dependent genes, rendering these particular targets more sensitive to changes in Ime1 levels but insensitive to changes in the affinity of Ime1 binding. An initial search for transcription factor motifs uncovered the Ume6 binding site in both sets of genes, but did not reveal any notable differences in the motif content of the two sets of target promoters (*P.S & B.A.C, unpublished data*). However, it remains possible that each set of promoters contains a unique combination of motifs and co-factors that control the allele-dependent response.

Finding consistent patterns among the hundreds of eQTL is a major challenge in the study of quantitative variation in gene expression [13]. Investigators have focused on *cis*-eQTL, the number of targets, or the effect size of a given eQTL as ways to screen eQTL for the variants most likely to be important. We find that that the fraction of variation in gene expression explained by the sporulation QTN is not predictive of the fraction of variation in phenotype that they explain. The results are surprising since all four QTN lie in known or putative transcriptional regulators and, therefore, must exert their phenotypic effects through changes in gene expression. It remains to be determined if this same trend will hold for causal genes that are not TFs. Perhaps the indirect effects of non-TFs on gene expression will better correlate with downstream phenotypes than the direct effects of TFs. However, early studies on laboratory-derived mutations showed that there were no significant differences between TFs and non-TFs in terms of their effects on gene expression [53]. Therefore, we suspect that our results will be applicable to naturally occurring polymorphisms in non-TFs as well. We have also not found any distinction between *cis*- and *trans*-QTN. While all four QTN act like eQTL “hot spots”, either *cis*- or *trans*-eQTL can can explain large proportions of the variation in gene expression (*RSF1c* and *IME1nc*) or in phenotype (*RME1nc* and *IME1c*). These results suggest that, along with the amount of gene expression variation explained by a given QTN, the identity and function of the particular genes affected may be important in identifying the eQTL that has the most significant role in controlling phenotypic variation.

## Materials and Methods

### Experimental design

The culture conditions for sporulation efficiency were modified from *Gerke et al.* [27] to accommodate larger samples for RNA-Seq preparations. Two replicates each of the 16 strains in the vineyard background allele replacement panel were grown for 14 hours at 30C in 96-well blocks containing 500 ul of Yeast Peptone Dextrose (YPD) medium with 2% dextrose. The replicates were pooled and diluted 1∶50 into 250 ml conical flasks containing 50 ml of 1% potassium acetate to induce sporulation. Cultures were grown for 30 hours and sporulation efficiencies were measured as described in Gerke et al. [27]. The entire procedure was repeated on different days until we had four biological replicates for each strain.

For RNA-Seq, cultures were grown as described above but growth was stopped after 2 hours in potassium acetate by spinning cells down and freezing the cell pellets at −80°C. Cells were harvested at this stage and total RNA was extracted [20]. The entire procedure including total RNA extraction was repeated on different days until we had four biological replicates for each strain.

mRNA was extracted with the DynaI mRNA DIRECT kit (Life Technologies) and fragmented with a Covaris Focused ultrasonicator. mRNA extraction and fragmentation, random hexamer priming of cDNA and Illumina library preparations were done by the Genome Technology Access Center (GTAC) at Washington University in St. Louis (https://gtac.wustl.edu) using standard procedures [54]. The liquid handling steps from the mRNA extraction stage onwards were performed on all 64 samples simultaneously using the Caliper Sciclone Automated Liquid Handling Workstation (PerkinElmer).

### RNA-seq

Illumina libraries were prepared from the cDNA of each of the 64 samples. We obtained libraries from all the samples except the strain with vineyard alleles of *RME1nc*, *RSF1c*, *IME1nc* and oak allele of *IME1c* which had only 3 replicates for the subsequent analyses. The libraries were indexed separately and pooled into one sequencing reaction. The pool was run on multiple lanes until we obtained a minimum of 4 million reads per sample. The sequencing reads for each sample were combined across all sequencing runs. If present, adapter dimers were removed and the sequencing reads were aligned to the Verified and Uncharacterized open reading frames (ORFs) in the *S. cerevisiae* reference genome (S288C, genome release R63-1-1, Saccharomyces Genome Database (SGD, http://www.yeastgenome.org/)) using Bowtie, version 0.12.7 [55]. Only unique alignments with maximum 2 mismatches in the –best alignment mode were accepted. The counts for all the reads aligned to a given ORF were summed to give the raw counts per ORF. The raw counts were scaled to account for differences in sequencing depths per sample by calculating the normalized count values across all samples as described in DESeq, version 1.9.11 [56]. To normalize samples, the ratio of a gene's counts to its geometric mean across all the samples was calculated for each gene. Assuming that most genes are not differentially expressed, the scaling factor for each sample was the median of the ratios of all the genes in the sample. For each gene in a given sample, the counts were then normalized by the scaling factor for that sample. The normalized gene counts were used for all further analyses. The lowest 20^th^ percentile of ORFs, based on the sum of the normalized counts across all samples for the given ORF, was removed to reduce the number of tested hypotheses and false positives. 4633 ORFs out of the initial 5792 ORFs remained after the filtering stage.

The normalized gene counts and the raw expression data discussed in this publication have been deposited in NCBI's Gene Expression Omnibus [57] and are accessible through GEO Series accession number GSE55409 (http://www.ncbi.nlm.nih.gov/geo/query/acc.cgi?acc=GSE55409).

### Statistical analyses

All statistical analyses were performed in R [58]. Linear regression was performed using the lm function in R. The genes whose expression is best explained by the genotype of the sporulation QTN were found in a two-step process. To eliminate any variation due to growing the allele replacement panel on different days, for a given ORF, i, we computed the residual gene expression (ε_i_) for all 4633 ORFs after removing the additive effect of the day of growth (DAY) on the normalized counts (N_i_) of each ORF. Thus, the DAY model was applied on a gene-by-gene basis resulting in 4633 gene-specific DAY models.
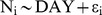



The residual gene expression from the DAY models was used in subsequent analyses. For each gene, the effect of the sporulation QTN on gene expression was computed in a second linear model by regressing the genotype of each of the four QTN (*RME1nc*, *RSF1c*, *IME1c* and *IME1nc*) on the residual gene expression from the previous modeling step. Again, 4633 gene-specific expression models were run. The 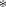 in the model below indicates that both additive and interaction effects were considered.
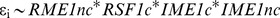



The effect of the sporulation QTN on gene expression was also compared to results from alternative model where the effect of DAY as well as the genotype of each of the four QTN (*RME1nc*, *RSF1c*, *IME1c* and *IME1nc*) was regressed on the log-transformed normalized expression counts of each gene.
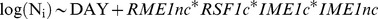



We used the Benjamini-Hochberg procedure [59] on the model p-values to control the False Discovery Rate (FDR) to 10% and obtained 289 significant models. The unadjusted p-value of the significant models was 0.006 or lower. We also assessed significance of the gene expression models by permuting the genotype designations on all 63 samples and regressing the effect of the permuted genotype on the residual expression from the DAY model for all ORFs. The p-value corresponding to the lowest 5^th^ percentile was obtained from the distribution of model p-values across the genome. The permutations and genotype modeling were repeated 1000 times to determine the distribution of the 5^th^ percentile of model p-values. We found the unadjusted p-value threshold from the FDR control to be almost 2 standard deviations below the average of the distribution of 5^th^ percentile model p-values obtained from the permutations. Since the FDR p-value threshold was more stringent than that obtained from the permutations above, we performed the remaining analyses on the 289 significant models.

The effect of individual QTN on gene expression was found by comparing nested models using ANOVA and calculating the fraction of variance explained by all significant factors of the given allele. In the ANOVA analysis, individual factors were considered to be statistically significant with a fairly permissive threshold (f-statistic p-value<0.1). We chose to report the effect of each variant as the computed variance explained by each variant, rather than the magnitude of the regression coefficients. We chose this metric because genes are expressed on very different scales which makes it difficult to interpret effect sizes across genes.

The coefficient of variation (CV = σ/μ) of the expression of each ORF across all four biological replicates was calculated for all 5792 ORFs in the genome. For a given ORF, σ represents the standard deviation of gene expression counts across the four biological replicates and μ represents the mean of gene expression counts across the biological replicates. To remove the effect of day of growth and to perform this particular analysis on the original expression scale, the normalized expression counts (using the DESeq normalization procedure) for each gene were further normalized for day-to-day variation as follows. A given day was arbitrarily chosen as Day A. For each ORF, the fitted values from the DAY model for all samples grown on a given day represent the mean expression of the ORF across all 16 strains in the panel for the given day. Variation due to the growing the allele replacement panel on different days was removed by dividing N_i_, the normalized gene expression counts for the ORF by the ratio of the mean expression of the particular ORF on a given day to the mean expression of that ORF in the 16 strains grown on day A. These “day-corrected” expression values were used for the CV calculations as well as for the heat map (Figure S1).

The wilcoxon rank sum test was applied using the standard *wilcox.test* function in R [58]. Enrichment analysis for gene-ontology (GO) categories was performed using the functional category analysis tools at DAVID Bioinformatics Resource 6.7 [60], [61].

## Supporting Information

Figure S1 Expression profiles of the genes significantly affected by the sporulation QTN. The expression profiles of the 289 genes with significant gene expression models are shown. All 16 genotypes are represented by the columns (x-axis) while the rows (y-axis) represent hierarchically clustered z-scores of gene expression of each gene across all 16 genotypes. Each expression value is the mean expression of the gene in the given genotype across four replicates using the residual expression of the gene after removing the effect of the day of growth. The only exception is the strain with vineyard alleles of *RME1nc*, *RSF1c*, *IME1nc* and oak allele of *IME1c* which only had three replicates. The genotypes of each strain are shown below the heatmap where ‘O’ represents the oak allele and ‘W’ represents the vineyard allele. The mean sporulation efficiencies (%) from four replicates of each strain in the allele replacement panel are also shown.
(TIF)

Figure S2 Comparison of linear models of the effect of genotype on gene expression using log-transformed and untransformed expression values. a. Histograms comparing *R^2^* values obtained for linear models of gene expression using log-transformed *(red)* and untransformed *(blue)* expression data for all 5792 genes in the genome. The *R^2^* values obtained *(x-axis)* and the numbers of models with the particular *R^2^* value *(y-axis)* are shown. b. Scatter plot comparing the *R^2^* values obtained for linear models using untransformed *(x-axis)* and log-transformed *(y-axis)* expression data for the 289 genes with significant expression models using untransformed expression data. The blue lines represent the *R^2^* value for the sporulation efficiency model.
(TIF)

Table S1 Sporulation efficiencies of allele replacement panel strains.
(XLSX)

Table S2 Effect of sporulation QTN on sporulation efficiency.
(DOCX)

Table S3 Effect of sporulation QTN on gene expression.
(XLSX)

Table S4 R-squared values for the expression models for genes significantly affected by the sporulation QTN.
(XLSX)

## References

[1] Stranger BE, Stahl EA, Raj T (2011) Progress and promise of genome-wide association studies for human complex trait genetics. Genetics 187: 367–383. 21115973 10.1534/genetics.110.120907 PMC3030483

[2] Liti G, Louis EJ (2012) Advances in quantitative trait analysis in yeast. PLoS Genet 8: e1002912. 22916041 10.1371/journal.pgen.1002912 PMC3420948

[3] Flint J, Mackay TF (2009) Genetic architecture of quantitative traits in mice, flies, and humans. Genome Res 19: 723–733. 19411597 10.1101/gr.086660.108 PMC3647534

[4] Maurano MT, Humbert R, Rynes E, Thurman RE, Haugen E, et al (2012) Systematic localization of common disease-associated variation in regulatory DNA. Science 337: 1190–1195. 22955828 10.1126/science.1222794 PMC3771521

[5] Brem RB, Yvert G, Clinton R, Kruglyak L (2002) Genetic dissection of transcriptional regulation in budding yeast. Science 296: 752–755. 11923494 10.1126/science.1069516

[6] Cheung VG, Conlin LK, Weber TM, Arcaro M, Jen KY, et al (2003) Natural variation in human gene expression assessed in lymphoblastoid cells. Nat Genet 33: 422–425. 12567189 10.1038/ng1094

[7] Brem RB, Kruglyak L (2005) The landscape of genetic complexity across 5,700 gene expression traits in yeast. Proc Natl Acad Sci U S A 102: 1572–1577. 15659551 10.1073/pnas.0408709102 PMC547855

[8] Gagneur J, Stegle O, Zhu C, Jakob P, Tekkedil MM, et al (2013) Genotype-environment interactions reveal causal pathways that mediate genetic effects on phenotype. PLoS Genet 9: e1003803. 24068968 10.1371/journal.pgen.1003803 PMC3778020

[9] Smith EN, Kruglyak L (2008) Gene-environment interaction in yeast gene expression. PLoS Biol 6: e83. 18416601 10.1371/journal.pbio.0060083 PMC2292755

[10] Yvert G, Brem RB, Whittle J, Akey JM, Foss E, et al (2003) Trans-acting regulatory variation in Saccharomyces cerevisiae and the role of transcription factors. Nat Genet 35: 57–64. 12897782 10.1038/ng1222

[11] Nica AC, Dermitzakis ET (2013) Expression quantitative trait loci: present and future. Philos Trans R Soc Lond B Biol Sci 368: 20120362. 23650636 10.1098/rstb.2012.0362 PMC3682727

[12] Gilad Y, Rifkin SA, Pritchard JK (2008) Revealing the architecture of gene regulation: the promise of eQTL studies. Trends Genet 24: 408–415. 18597885 10.1016/j.tig.2008.06.001 PMC2583071

[13] Majewski J, Pastinen T (2011) The study of eQTL variations by RNA-seq: from SNPs to phenotypes. Trends Genet 27: 72–79. 21122937 10.1016/j.tig.2010.10.006

[14] Jansen RC, Nap JP (2001) Genetical genomics: the added value from segregation. Trends Genet 17: 388–391. 11418218 10.1016/s0168-9525(01)02310-1

[15] Curtis C, Shah SP, Chin SF, Turashvili G, Rueda OM, et al (2012) The genomic and transcriptomic architecture of 2,000 breast tumours reveals novel subgroups. Nature 486: 346–352. 22522925 10.1038/nature10983 PMC3440846

[16] Nicolae DL, Gamazon E, Zhang W, Duan S, Dolan ME, et al (2010) Trait-associated SNPs are more likely to be eQTLs: annotation to enhance discovery from GWAS. PLoS Genet 6: e1000888. 20369019 10.1371/journal.pgen.1000888 PMC2848547

[17] Li Q, Seo JH, Stranger B, McKenna A, Pe'er I, et al (2013) Integrative eQTL-based analyses reveal the biology of breast cancer risk loci. Cell 152: 633–641. 23374354 10.1016/j.cell.2012.12.034 PMC4165609

[18] Grundberg E, Small KS, Hedman AK, Nica AC, Buil A, et al (2012) Mapping cis- and trans-regulatory effects across multiple tissues in twins. Nat Genet 44: 1084–1089. 22941192 10.1038/ng.2394 PMC3784328

[19] Cookson W, Liang L, Abecasis G, Moffatt M, Lathrop M (2009) Mapping complex disease traits with global gene expression. Nat Rev Genet 10: 184–194. 19223927 10.1038/nrg2537 PMC4550035

[20] Gerke JP, Chen CT, Cohen BA (2006) Natural isolates of Saccharomyces cerevisiae display complex genetic variation in sporulation efficiency. Genetics 174: 985–997. 16951083 10.1534/genetics.106.058453 PMC1602093

[21] Honigberg SM, Purnapatre K (2003) Signal pathway integration in the switch from the mitotic cell cycle to meiosis in yeast. J Cell Sci 116: 2137–2147. 12730290 10.1242/jcs.00460

[22] Kassir Y, Adir N, Boger-Nadjar E, Raviv NG, Rubin-Bejerano I, et al (2003) Transcriptional regulation of meiosis in budding yeast. Int Rev Cytol 224: 111–171. 12722950 10.1016/s0074-7696(05)24004-4

[23] Vershon AK, Pierce M (2000) Transcriptional regulation of meiosis in yeast. Curr Opin Cell Biol 12: 334–339. 10801467 10.1016/s0955-0674(00)00104-6

[24] Deutschbauer AM, Davis RW (2005) Quantitative trait loci mapped to single-nucleotide resolution in yeast. Nat Genet 37: 1333–1340. 16273108 10.1038/ng1674

[25] Ben-Ari G, Zenvirth D, Sherman A, David L, Klutstein M, et al (2006) Four linked genes participate in controlling sporulation efficiency in budding yeast. PLoS Genet 2: e195. 17112318 10.1371/journal.pgen.0020195 PMC1636695

[26] Tomar P, Bhatia A, Ramdas S, Diao L, Bhanot G, et al (2013) Sporulation genes associated with sporulation efficiency in natural isolates of yeast. PLoS One 8: e69765. 23874994 10.1371/journal.pone.0069765 PMC3714247

[27] Gerke J, Lorenz K, Cohen B (2009) Genetic interactions between transcription factors cause natural variation in yeast. Science 323: 498–501. 19164747 10.1126/science.1166426 PMC4984536

[28] Gerke J, Lorenz K, Ramnarine S, Cohen B (2010) Gene-environment interactions at nucleotide resolution. PLoS Genet 6: e1001144. 20941394 10.1371/journal.pgen.1001144 PMC2947989

[29] Smith HE, Driscoll SE, Sia RA, Yuan HE, Mitchell AP (1993) Genetic evidence for transcriptional activation by the yeast IME1 gene product. Genetics 133: 775–784. 8462841 10.1093/genetics/133.4.775 PMC1205399

[30] Rubin-Bejerano I, Mandel S, Robzyk K, Kassir Y (1996) Induction of meiosis in Saccharomyces cerevisiae depends on conversion of the transcriptional represssor Ume6 to a positive regulator by its regulated association with the transcriptional activator Ime1. Mol Cell Biol 16: 2518–2526. 8628320 10.1128/mcb.16.5.2518 PMC231241

[31] Covitz PA, Mitchell AP (1993) Repression by the yeast meiotic inhibitor RME1. Genes Dev 7: 1598–1608. 8339935 10.1101/gad.7.8.1598

[32] Lu L, Roberts G, Simon K, Yu J, Hudson AP (2003) Rsf1p, a protein required for respiratory growth of Saccharomyces cerevisiae. Curr Genet 43: 263–272. 12734673 10.1007/s00294-003-0398-z

[33] Roberts GG 3rd, Hudson AP (2009) Rsf1p is required for an efficient metabolic shift from fermentative to glycerol-based respiratory growth in S. cerevisiae. Yeast 26: 95–110. 19235764 10.1002/yea.1655

[34] Jambhekar A, Amon A (2008) Control of meiosis by respiration. Curr Biol 18: 969–975. 18595705 10.1016/j.cub.2008.05.047 PMC2504020

[35] Mitchell AP, Driscoll SE, Smith HE (1990) Positive control of sporulation-specific genes by the IME1 and IME2 products in Saccharomyces cerevisiae. Mol Cell Biol 10: 2104–2110. 2183020 10.1128/mcb.10.5.2104-2110.1990 PMC360558

[36] Wang Z, Gerstein M, Snyder M (2009) RNA-Seq: a revolutionary tool for transcriptomics. Nat Rev Genet 10: 57–63. 19015660 10.1038/nrg2484 PMC2949280

[37] Levin JZ, Yassour M, Adiconis X, Nusbaum C, Thompson DA, et al (2010) Comprehensive comparative analysis of strand-specific RNA sequencing methods. Nat Methods 7: 709–715. 20711195 10.1038/nmeth.1491 PMC3005310

[38] Toung JM, Morley M, Li M, Cheung VG (2011) RNA-sequence analysis of human B-cells. Genome Res 21: 991–998. 21536721 10.1101/gr.116335.110 PMC3106332

[39] Andersen G, Bjornberg O, Polakova S, Pynyaha Y, Rasmussen A, et al (2008) A second pathway to degrade pyrimidine nucleic acid precursors in eukaryotes. J Mol Biol 380: 656–666. 18550080 10.1016/j.jmb.2008.05.029

[40] Deng C, Saunders WS (2001) RIM4 encodes a meiotic activator required for early events of meiosis in Saccharomyces cerevisiae. Mol Genet Genomics 266: 497–504. 11713679 10.1007/s004380100571

[41] Garcia-Alvarez N, Teichert U, Wolf DH (1987) Proteinase yscD mutants of yeast. Isolation and characterization. Eur J Biochem 163: 339–346. 3545833 10.1111/j.1432-1033.1987.tb10805.x

[42] Smith HE, Su SS, Neigeborn L, Driscoll SE, Mitchell AP (1990) Role of IME1 expression in regulation of meiosis in Saccharomyces cerevisiae. Mol Cell Biol 10: 6103–6113. 2247050 10.1128/mcb.10.12.6103-6113.1990 PMC362885

[43] Cheung VG, Spielman RS (2009) Genetics of human gene expression: mapping DNA variants that influence gene expression. Nat Rev Genet 10: 595–604. 19636342 10.1038/nrg2630 PMC2989458

[44] Orozco LD, Bennett BJ, Farber CR, Ghazalpour A, Pan C, et al (2012) Unraveling inflammatory responses using systems genetics and gene-environment interactions in macrophages. Cell 151: 658–670. 23101632 10.1016/j.cell.2012.08.043 PMC3513387

[45] Rockman MV, Kruglyak L (2006) Genetics of global gene expression. Nat Rev Genet 7: 862–872. 17047685 10.1038/nrg1964

[46] Waddington CH (1942) Canalization of development and the inheritance of acquired characters. Nature 150: 563–565. 10.1038/1831654a0 13666847

[47] Arias AM, Hayward P (2006) Filtering transcriptional noise during development: concepts and mechanisms. Nat Rev Genet 7: 34–44. 16369570 10.1038/nrg1750

[48] Fu J, Keurentjes JJ, Bouwmeester H, America T, Verstappen FW, et al (2009) System-wide molecular evidence for phenotypic buffering in Arabidopsis. Nat Genet 41: 166–167. 19169256 10.1038/ng.308

[49] Raj A, Rifkin SA, Andersen E, van Oudenaarden A (2010) Variability in gene expression underlies incomplete penetrance. Nature 463: 913–918. 20164922 10.1038/nature08781 PMC2836165

[50] Clemmons AW, Wasserman SA (2013) Combinatorial effects of transposable elements on gene expression and phenotypic robustness in Drosophila melanogaster development. G3 (Bethesda) 3: 1531–1538. 23833214 10.1534/g3.113.006791 PMC3755913

[51] Rubinstein A, Gurevich V, Kasulin-Boneh Z, Pnueli L, Kassir Y, et al (2007) Faithful modeling of transient expression and its application to elucidating negative feedback regulation. Proc Natl Acad Sci U S A 104: 6241–6246. 17400752 10.1073/pnas.0611168104 PMC1851052

[52] Anderson SF, Steber CM, Esposito RE, Coleman JE (1995) UME6, a negative regulator of meiosis in Saccharomyces cerevisiae, contains a C-terminal Zn2Cys6 binuclear cluster that binds the URS1 DNA sequence in a zinc-dependent manner. Protein Sci 4: 1832–1843. 8528081 10.1002/pro.5560040918 PMC2143208

[53] Hughes TR, Marton MJ, Jones AR, Roberts CJ, Stoughton R, et al (2000) Functional discovery via a compendium of expression profiles. Cell 102: 109–126. 10929718 10.1016/s0092-8674(00)00015-5

[54] Wilhelm BT, Landry JR (2009) RNA-Seq-quantitative measurement of expression through massively parallel RNA-sequencing. Methods 48: 249–257. 19336255 10.1016/j.ymeth.2009.03.016

[55] Langmead B, Trapnell C, Pop M, Salzberg SL (2009) Ultrafast and memory-efficient alignment of short DNA sequences to the human genome. Genome Biol 10: R25. 19261174 10.1186/gb-2009-10-3-r25 PMC2690996

[56] Anders S, Huber W (2010) Differential expression analysis for sequence count data. Genome Biol 11: R106. 20979621 10.1186/gb-2010-11-10-r106 PMC3218662

[57] Edgar R, Domrachev M, Lash AE (2002) Gene Expression Omnibus: NCBI gene expression and hybridization array data repository. Nucleic Acids Res 30: 207–210. 11752295 10.1093/nar/30.1.207 PMC99122

[58] Team RC (2012) R: A language and environment for statistical computing. R Foundation for Statistical Computing, Vienna, Austria.

[59] Benjamini Y, Hochberg Y (1995) Controlling the false discovery rate: a practical and powerful approach to multiple testing. Journal of the Royal Statistical Society Series B 57: 289–300.

[60] Huang da W, Sherman BT, Lempicki RA (2009) Bioinformatics enrichment tools: paths toward the comprehensive functional analysis of large gene lists. Nucleic Acids Res 37: 1–13. 19033363 10.1093/nar/gkn923 PMC2615629

[61] Huang da W, Sherman BT, Zheng X, Yang J, Imamichi T, et al (2009) Extracting biological meaning from large gene lists with DAVID. Curr Protoc Bioinformatics Chapter 13: Unit 13 11. 19728287 10.1002/0471250953.bi1311s27

