# Supplementary figures and images for: Single Nucleotide Variants in Transcription Factors Associate More Tightly with Phenotype than with Gene Expression
Source: PLoS Genet. 2014 May 1;10(5):e1004325. doi: 10.1371/journal.pgen.1004325 (PMC4006743; doi:10.1371/journal.pgen.1004325)

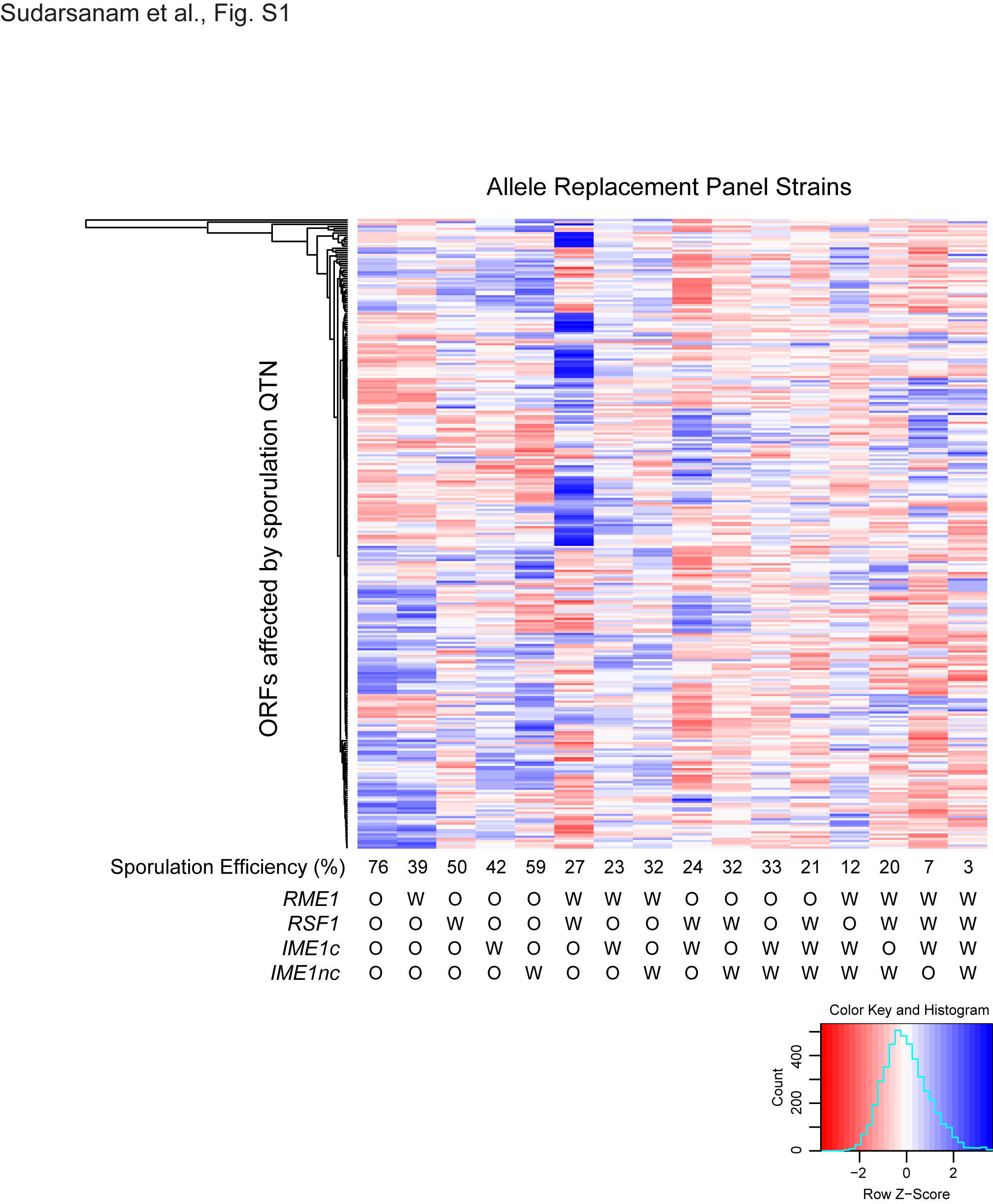

Supplement: Figure S1 — Expression profiles of the genes significantly affected by the sporulation QTN. The expression profiles of the 289 genes with significant gene expression models are shown. All 16 genotypes are represented by the columns (x-axis) while the rows (y-axis) represent hierarchically clustered z-scores of gene expression of each gene across all 16 genotypes. Each expression value is the mean expression of the gene in the given genotype across four replicates using the residual expression of the gene after removing the effect of the day of growth. The only exception is the strain with vineyard alleles of RME1nc, RSF1c, IME1nc and oak allele of IME1c which only had three replicates. The genotypes of each strain are shown below the heatmap where ‘O’ represents the oak allele and ‘W’ represents the vineyard allele. The mean sporulation efficiencies (%) from four replicates of each strain in the allele replacement panel are also shown. (TIF) [file pgen.1004325.s001.tif]

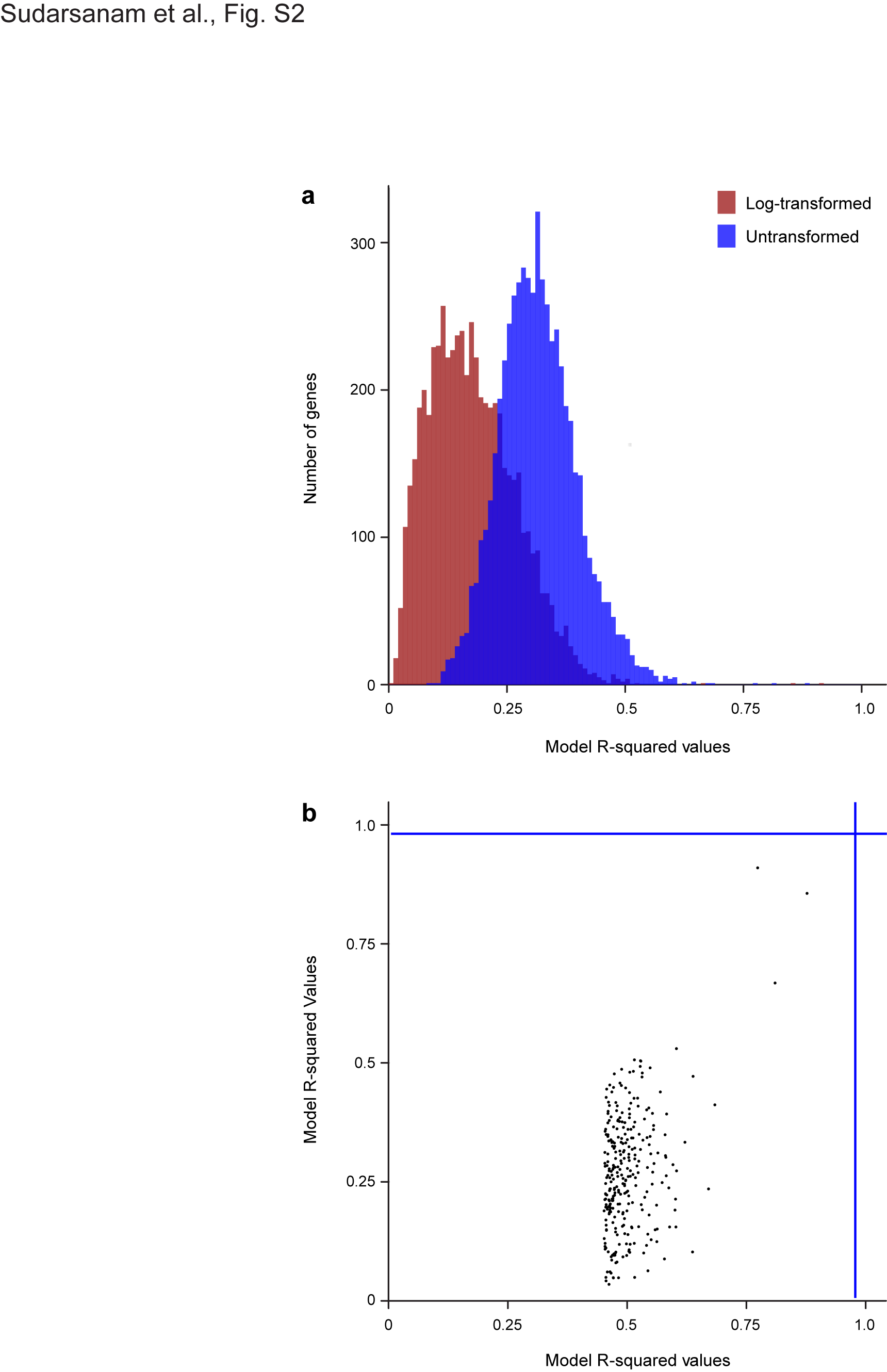

Supplement: Figure S2 — Comparison of linear models of the effect of genotype on gene expression using log-transformed and untransformed expression values. a. Histograms comparing R2 values obtained for linear models of gene expression using log-transformed (red) and untransformed (blue) expression data for all 5792 genes in the genome. The R2 values obtained (x-axis) and the numbers of models with the particular R2 value (y-axis) are shown. b. Scatter plot comparing the R2 values obtained for linear models using untransformed (x-axis) and log-transformed (y-axis) expression data for the 289 genes with significant expression models using untransformed expression data. The blue lines represent the R2 value for the sporulation efficiency model. (TIF) [file pgen.1004325.s002.tif]
