# Supplementary material for: Single Nucleotide Variants in Transcription Factors Associate More Tightly with Phenotype than with Gene Expression
Source: PLoS Genet. 2014 May 1;10(5):e1004325. doi: 10.1371/journal.pgen.1004325 (PMC4006743; doi:10.1371/journal.pgen.1004325)
Supplement: Table S2 — Effect of sporulation QTN on sporulation efficiency. (DOCX) [file pgen.1004325.s004.docx]

**SUPPLEMENTARY INFORMATION**

**TABLES**

**S2. Effect of sporulation QTN on sporulation efficiency.** All experiments were performed in the vineyard strain background. The four sporulation QTN are: *RME1nc*: *RME1(indel-308A)*, *RSF1c*: *RSF1(D181G)*, *IME1c*: *IME1(L325M)*, *IME1nc*: *IME1(A-548G)*. Effects and standard errors denote sporulation efficiency values relative to the vineyard converted strain containing all four oak alleles in the vineyard background which sporulates at 76.2%. P-values ≤ 0.05 are in bold.

| Coefficient (Allele replacements) | Estimated Effect | Standard Error | t value | | P value |
| --- | --- | --- | --- | --- | --- |
| *RME1nc* | -37.1 | 2.11 | -17.56 | **< 2e-16** | |
| *RSF1c* | -26.5 | 2.11 | -12.55 | **< 2e-16** | |
| *IME1c* | -34.3 | 2.11 | -16.26 | **< 2e-16** | |
| *IME1nc* | -17.1 | 2.11 | -8.08 | **1.64e-10** | |
| *RME1nc*RSF1c* | 14.0 | 2.99 | 4.69 | **2.28e-05** | |
| *RME1nc*IME1c* | 18.1 | 2.99 | 6.07 | **1.97e-07** | |
| *RME1nc*IME1nc* | 9.5 | 2.99 | 3.19 | **0.003** | |
| *RSF1c*IME1c* | 9.0 | 2.99 | 3.01 | **0.004** | |
| *RSF1c*IME1nc* | -0.99 | 2.99 | -0.33 | 0.742 | |
| *IME1c*IME1nc* | 8.6 | 2.99 | 2.88 | **0.006** | |
| *RME1nc*RSF1c*IME1c* | -13.0 | 4.22 | -3.07 | **0.004** | |
| *RME1nc*RSF1c*IME1nc* | 1.8 | 4.22 | 0.43 | 0.667 | |
| *RME1nc*IME1c*IME1nc* | -11.9 | 4.22 | -2.83 | **0.007** | |
| *RSF1c*IME1c*IME1nc* | 5.62 | 4.22 | 1.33 | 0.190 | |
| *RME1nc*RSF1c*IME1c* IME1nc* | 0.53 | 5.97 | 0.088 | 0.930 | |

***Multiple R-squared = 0.98; F-statistic: 164.7 on 15 and 48 DF, p-value <2.2e-16***
